# Quantifying the complexity and similarity of chess openings using online chess community data

**DOI:** 10.1038/s41598-023-31658-w

**Published:** 2023-04-01

**Authors:** Giordano De Marzo, Vito D. P. Servedio

**Affiliations:** 1 Centro Ricerche Enrico Fermi, Piazza del Viminale, 1, 00184 Rome, Italy; 2 Physics Department, Sapienza University of Rome, P.le A. Moro, 2, 00185 Rome, Italy; 3 Sapienza School for Advanced Studies, “Sapienza”, P.le A. Moro, 2, 00185 Rome, Italy; 4 Complexity Science Hub Vienna, Josefstädter Straße 39, Vienna, 1080 Austria

**Keywords:** Complex networks, Applied physics

## Abstract

Chess is a centuries-old game that continues to be widely played worldwide. Opening Theory is one of the pillars of chess and requires years of study to be mastered. In this paper, we use the games played in an online chess platform to exploit the “wisdom of the crowd” and answer questions traditionally tackled only by chess experts. We first define a relatedness network of chess openings that quantifies how similar two openings are to play. Using this network, we identify communities of nodes corresponding to the most common opening choices and their mutual relationships. Furthermore, we demonstrate how the relatedness network can be used to forecast future openings players will start to play, with back-tested predictions outperforming a random predictor. We then apply the Economic Fitness and Complexity algorithm to measure the difficulty of openings and players’ skill levels. Our study not only provides a new perspective on chess analysis but also opens the possibility of suggesting personalized opening recommendations using complex network theory.

## Introduction

Chess has captivated countless individuals since its inception in the 6th century. Today, 1500 years later, it boasts over 600 million regular players worldwide (https://www.un.org/en/observances/world-chess-day). Considered by many to be one of the noblest intellectual arts, chess has played a significant role in human history, including the competition for intellectual supremacy between the Soviet Union and the United States, as seen in the 1972 world championship match between Bobby Fisher and Boris Spassky. Additionally, the iconic match between world champion Garry Kasparov and IBM supercomputer Deep Blue, won by the latter, established the superiority of the computer over the human mind in computational problems, marking a milestone in the history of artificial intelligence. Despite this, people have not lost interest in chess but instead have started using computers to further improve their understanding of the game.

The popularity of chess has also garnered attention from the scientific community, with early theoretical studies dating back to C. E. Shannon^1^. The advent of the internet and online chess platforms has allowed for the analysis of vast amounts of data using the tools of statistical physics and complex systems. For example, Refs.^2–4^ have shown that chess openings follow Heaps’ and Zipf’s laws, two statistical regularities that are often considered the footprint of complexity^5^. Chowdary et al.^6^ showed that skilled players can be distinguished based on their gaming behavior, and the opening diversity of players tends to decrease over time, with the emergence of individual playing styles. Other studies have focused on chess players’ ratings and their evolution^7,8^ as well as the popular level learning of the game^9^.

The vast number of different playable games, estimated by C. E. Shannon to be around $10^{120}$, is a key factor contributing to the complexity of chess. However, not all moves are equally good, and only a tiny fraction of the possible positions are observed in real games. The study of the sequence of initial good moves is known as Chess Opening Theory, with the Encyclopedia of Chess Openings (ECO) being the most authoritative resource on the subject, classifying openings into 500 different ECO codes^10^. The ECO groups are compiled by chess experts, mainly grandmasters, who select the most relevant lines and group them based on their expertise, making the process subjective and not data-driven. Openings are a central part of chess, and top-level players dedicate a significant portion of their time to studying new opening ideas and memorizing new lines. Mastering Opening Theory typically requires a deep knowledge of chess that is beyond the capabilities of most amateurs, and only world-level professional players have a complete understanding of the theory. However, we show that by leveraging complex network theory and considering a large community of chess players, it is possible to use the emerging social intelligence of the crowd (“the wisdom of the crowd”) to overcome this limitation. Although each player in the community has only partial knowledge, combining all their knowledge can provide a complete picture of the Opening Theory. This approach enables us to quantify chess features that only chess experts have been able to appreciate so far, such as the similarity between openings, the complexity of openings, and the quality of players’ opening repertoires.

## Results

### The bipartite network of chess players and openings

Network theory is one of the pillars of complexity since most complex systems spontaneously arrange into graphs, chess making no exception^11^. Bipartite networks, in particular, have received an increasing interest due to many systems displaying this peculiar graph arrangement. A graph is bipartite when there exist two classes of nodes such that the nodes of the same class do not connect to each other, while connections between the two classes are present. For example, one can represent the world trade network with the bipartite network given by the products and by the countries exporting them. By leveraging that network, it is possible to obtain state-of-the-art long-term GDP forecasts^12^ and to predict the industrial upgrading of countries^13,14^. We can identify a bipartite network structure also in chess and use it to gather novel insight into this game.

In technical terms, a bipartite network is a network whose nodes can be divided into two sets $\mathscr{P}$, $\mathscr{O}$ such that there are no links between nodes belonging to the same set. Denoting by *P* the number of nodes in the first set and by *O* the number of nodes in the second one, an unweighted directed bipartite network can be represented by a $P\times O$ matrix $\textbf{M}$ such that $M_{po}=1$ if $p\in P$ and $o\in O$ are connected and $M_{po}=0$ otherwise. In this study, we consider the bipartite network of chess players and chess openings, that we built using games played on the online chess platform lichess.org^15^ with a Blitz time control (see Data section). We chose Blitz games since this format is the most played online. The Lichess platform uses the *Glicko-2* system to rate players (see Data section). We consider the 500 chess openings with their ECO code as appearing in the “Encyclopedia of Chess Openings”^10,16^. Each ECO code corresponds to two nodes in the network since we distinguish between playing with White pieces or with Black pieces. For instance, if a game between player A with White and player B with Black falls under ECO code C20 (King’s pawn game), then A is connected to the opening C20W (King’s pawn game with White), while B to the opening C20B (King’s pawn game with Black). In this first part of the analysis, we selected a sub-sample of chess players with a rating above 2000 and who played at least 100 games with Black and 100 games with White during the period considered, from October 2015 to September 2016 (one year). This way, we ended up with a network composed of 2513 players and 982 openings (no player played 18 of the 1,000 openings during the time-laps we analyzed). The matrix *M* thus satisfies

$$\begin{aligned} M_{po}= {\left\{ \begin{array}{ll} 1 \ \text {if player}\ p \ \text {played opening}\ o\\ 0 \ \text {otherwise} \end{array}\right. } \end{aligned}$$

We can then define for each player *p* their diversification $d_p$ as the number of distinct openings they use $d_p=\sum _o^O M_{po}$ and for each opening *o* its ubiquity $u_o$ as the number of players who used it $u_o=\sum _p^P M_{po}$.

### The network of chess openings

**Figure 1:**
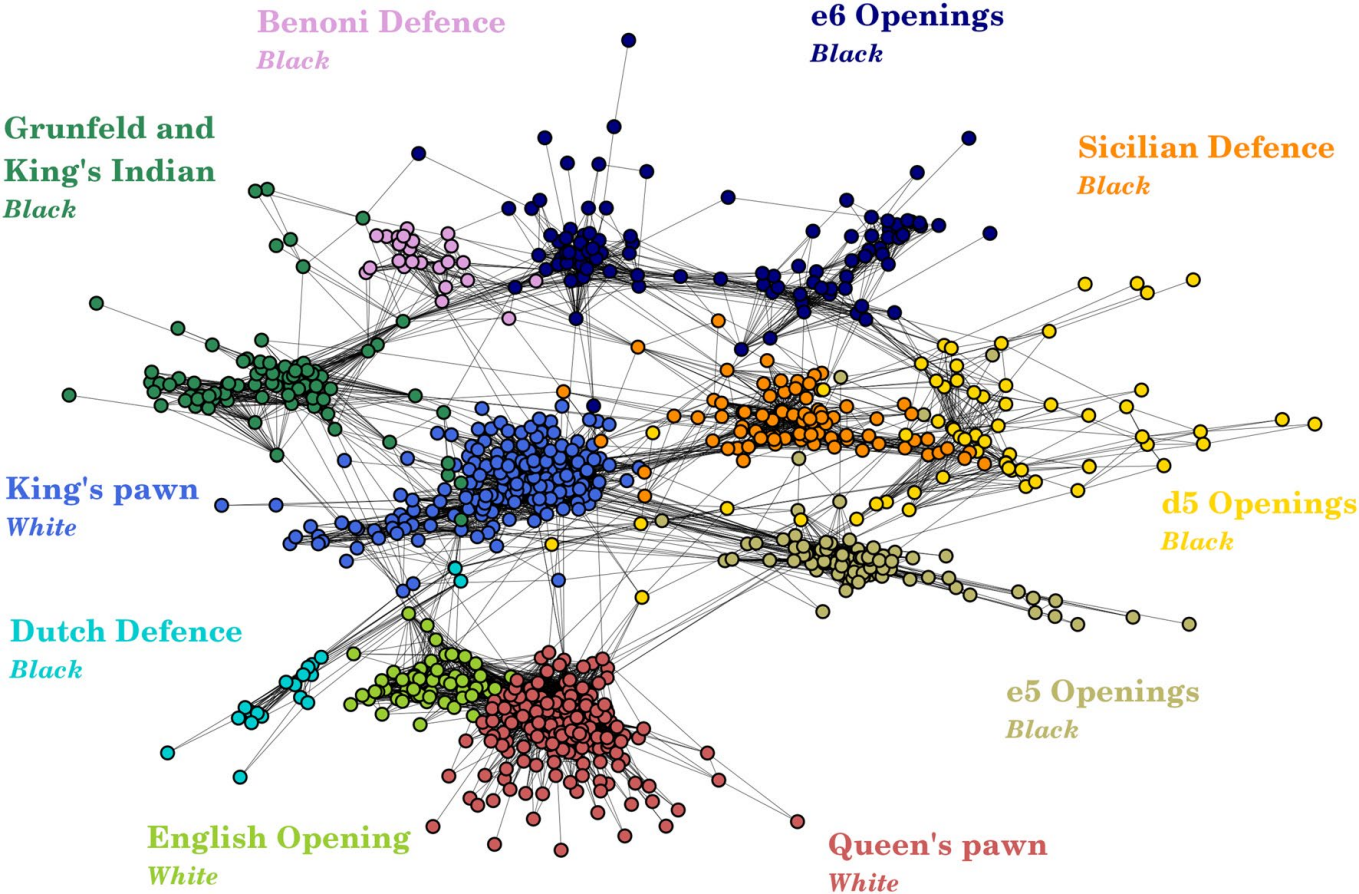
Network of openings. Relatedness network of openings obtained by projecting and validating the player-opening bipartite network. Openings close on this network are similar to play since they often appear together in players’ opening repertoires. Ten clusters identified using the Leiden community detection algorithm are represented in different colors, each cluster corresponding to a different opening choice.

**Figure 2:**
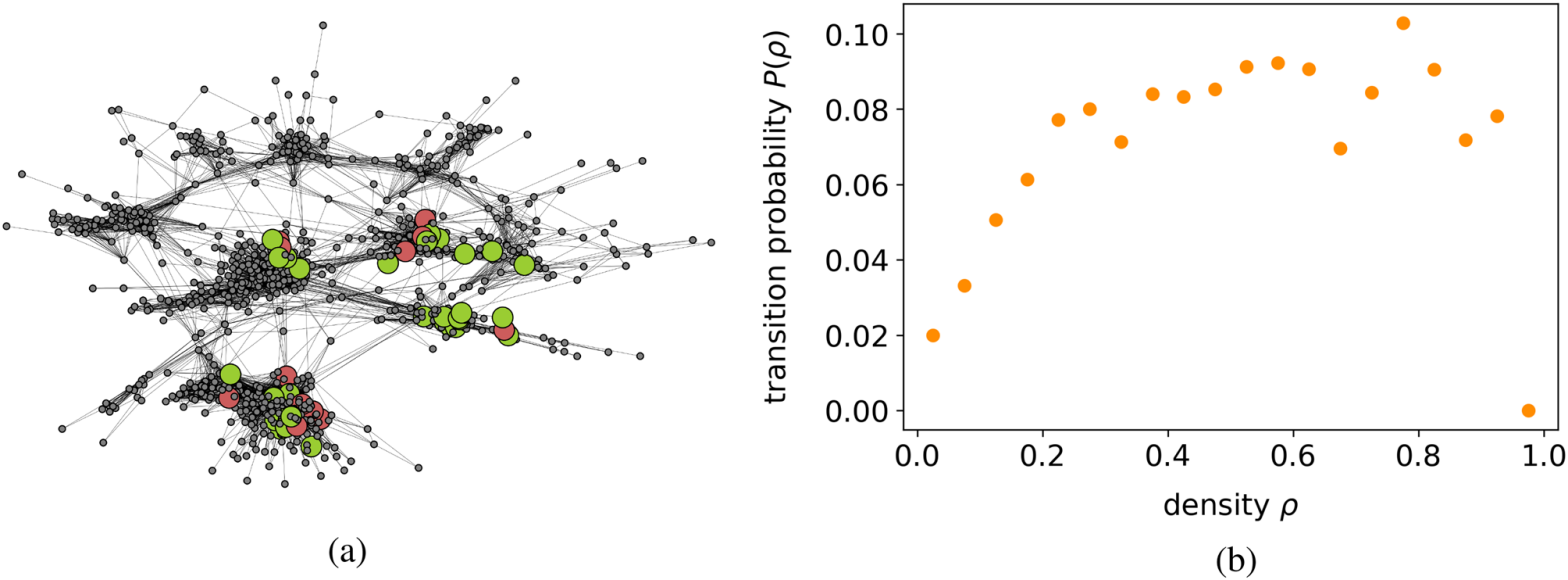
Prediction of future openings. (**a**) We plot, on the top of the opening network of Fig. 1, the openings a randomly selected player used during the period July 2016-September 2016 (green nodes) and the new openings he/she used in the period October 2016-December 2016 (red nodes). As it is possible to see, new openings are close to those openings the player already used. (**b**) Probability for a never used opening to start to be played as a function of its density, defined as the fraction of neighbour openings the player already uses. This probability is increasing in density, meaning players tend to learn openings close to those they already know. The network topology defines closeness.

Relatedness networks have been widely used in the economic complexity literature to quantify the similarity between nodes belonging to the same layer of bipartite networks^17^. For instance, considering the country-product network, the assumption is that two products require similar capabilities for being produced if they appear together in the export basket of many different countries or, in other words, if they co-occur often. In the same way, here we build the relatedness network of chess openings leveraging the idea that two of them are similar to play if many players play them both. We thus define the relatedness $W^*_{o_1 o_2}$ between two openings $o_1$, $o_2$ as

1 $$\begin{aligned} W^*_{o_1 o_2}=\sum _p^P M_{p o_1}M_{p o_2} \end{aligned}$$

However, the resulting matrix $\mathbf {W^*}$ contains many spurious co-occurrences. Two openings may often co-occur just because players with high diversification use them both by chance or because the openings are ubiquitous but not similar. In order to filter out such spurious co-occurrences, we exploit a null model, namely the Bipartite Configuration Model (BiCM)^18^. The idea is to retain only those links that can not be explained only through the ubiquity and the diversification of nodes and that are thus statistically significant; we report more details in Methods. In the following we denote by $\textbf{W}$ the matrix resulting from this procedure, whose elements satisfy

2 $$\begin{aligned} W_{o_1 o_2}= {\left\{ \begin{array}{ll} 1 \ \text {if}\ W^*_{o_1 o_2} \ \text {is statistically significant}\\ 0 \ \text {otherwise} \end{array}\right. } \end{aligned}$$

and we call relatedness network of openings, or simply network of openings, the network defined by such matrix. Since we obtain the network with a maximum entropy procedure, it is unbiased and its links connect openings that co-occur more often than they would in a random case.

We show the network of chess openings in Fig. 1. It is composed of 924 nodes out of the initial 982 since we filtered out isolated nodes and small components formed by pairs of nodes. We then applied the Leiden algorithm^19^ to this network to detect communities, that we indicate with different colours in the figure. There are ten clusters, three corresponding to openings played from the White perspective and the remaining seven to those played from the Black perspective. The three White clusters almost perfectly correspond to the three main choices White has as the first move: 1. e4 ($65\%$ of games), 1. d4 ($24\%$ of games), 1. c4 ($3\%$ of games) (The percentages come from the Lichess database https://lichess.org/analysis##0). More precisely,

- King’s pawn opening (light blue), where White’s first move consists in advancing the king’s pawn by two squares (1. e4)
- Queen’s pawn opening (red), characterized by White choosing as first move to advance the queen’s pawn by two squares (1. d4)
- English opening (light green), where White opens by moving the c2 pawn by two squares (1. c4). Also Reti opening (1. Nf6) is contained in this cluster since it often involves advancing the c2 pawn by two squares as second move (2. c4)

Also Black clusters nicely maps to Black’s main choices, but their number is higher since Black’s reply also depends on White’s first move. The clusters are

- e5 openings (khaki), where Black plays the move e5 as reply to a King’s pawn opening (1. e4 e5) or as reply to the English opening (1. c4 e5)
- e6 openings (dark blue), which can be divided in two main categories, as also evident from the shape of the cluster. The French opening (1. e4 e6) on the right, where e6 comes as reply to the King’s pawn opening, and Queen’s pawn game openings (Catalan, Bogo-Indian, Queen’s Indian and Nizmo-Indian) on the left, where Black plays e6 as second move after White having opened with her Queen’s pawn (1. d4 Nf6 2. c4 e6)
- Sicilian defense (orange), characterized by Black replying with c5 to White’s King’s pawn opening (1. e4 c5). This community also contains other openings characterized by Black playing c5 such as the Symmetric English (1. c4 c5).
- d5 openings (yellow), where d5 may comes as response to a Queen’s pawn opening (1. d4 d5) or to a King’s pawn opening as in the Caro-Kann defense (1. e4 c6 2. d4 d5)
- Benoni defense (Pink), that comes as response to White playing the Queen’s pawn opening (1. d4 Nf6 2. c4 c5)
- Grunfeld and King’s Indian defenses, both characterized by the same initial moves (1. d4 Nf6 2. c4 g6). It is worth remarking that both these openings are very similar to Benoni defense and this explains their closeness in the network
- Dutch defense, where Black responds with f5 to White’s Queen’s pawn opening (1. d4 f5)

We observe that clusters of White openings depend only on White’s first move, whereas those formed by Black openings also depend on the second move played by Black. It is worth noting that this clustered structure cannot be deduced directly from the ECO classification. In fact, openings belonging to the same ECO group split into different communities, and clusters are often formed by openings belonging to different ECO groups. Chess experts have a clear understanding of this clustered structure. Additionally, we ran the clustering algorithm multiple times to ensure the stability of the obtained structure. The results of different runs are almost the same. We observed only two possible clustering configurations, occurring roughly with the same frequency. The first configuration corresponds to Fig. 1, while the second one is slightly different: the French opening goes into the d5 openings cluster, while the rest of the e6 cluster gets merged with the Benoni cluster. The second clustering is also very reasonable since all French opening ECO codes include d5 as the second black move, while the Benoni involves playing e6 as the third move.

### Forecast of future openings

**Figure 3:**
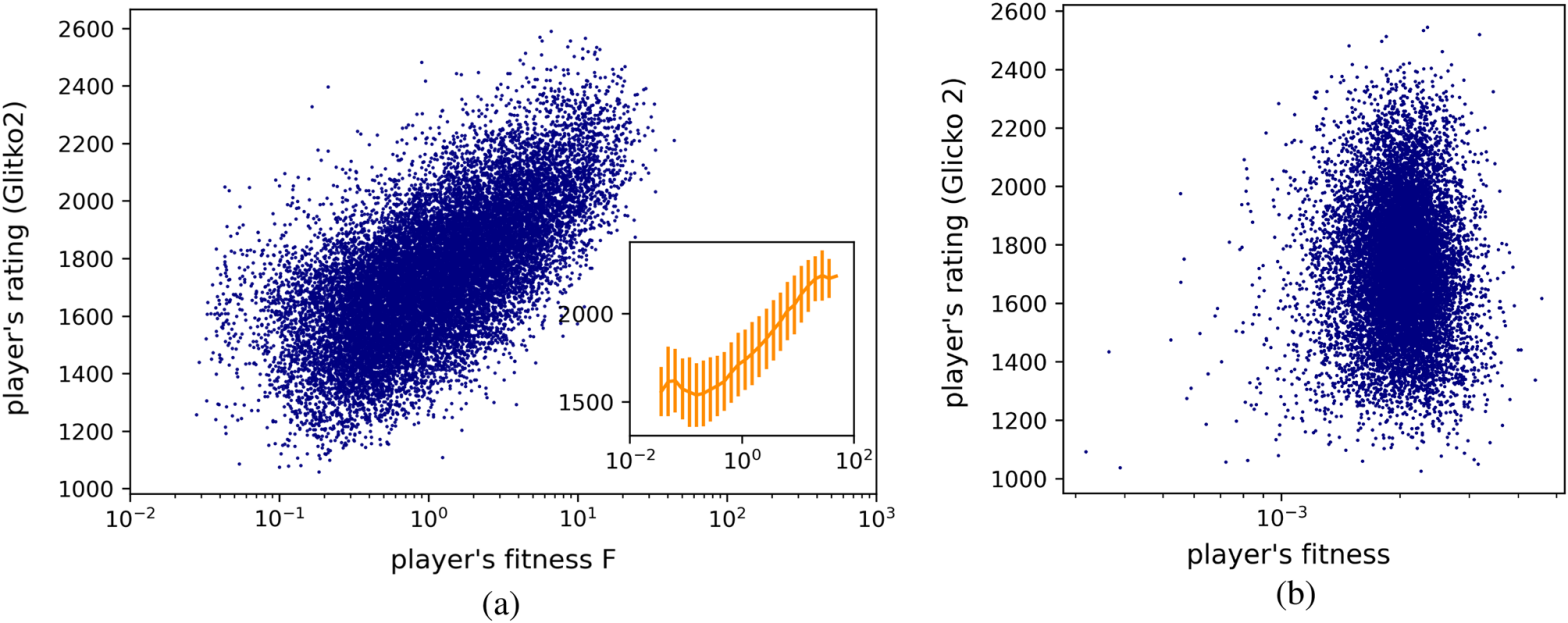
Fitness of players. (**a**) Comparison between players’ fitness and players’ Glicko2 rating. The Glicko2 score is assigned to chess players by Lichess.com, while fitness is an algorithmic measure of players’ opening ability, computed using ECO codes to identify openings. A strong correlation between the two quantities is observed, indicating that players’ opening preparation significantly affects their ability to win games and achieve high ratings. The binned average rating as a function of fitness is shown in the inset, with error bars determined by the standard deviation of the sample. (**b**) We compare fitness and rating using 6-plies to identify different openings. In this case, no correlation is observed between players’ fitness and rating.

As described in the preceding section, the relatedness of two openings measures their similarity in play, allowing the opening network to serve as a metric of distance between openings. While we previously inferred the clustered structure of the network qualitatively, we can now take an additional step and demonstrate that the opening network is capable of predicting a player’s future opening choices. This prediction is based on the idea that openings that are in close proximity to a player’s existing repertoire are more readily learned. This concept is illustrated in Fig. 2a, where we depict in green the openings a randomly selected player employed from July to September 2016, in red the openings she began using in the subsequent three months, and in small gray dots all the other openings that the player did not utilize. We observe that the new openings are similar to those used in the first time interval, giving rise to an ”adjacent possible” effect^20,21^. The adoption of a new opening enables players to explore other openings connected to it, and the learning of Opening Theory can be viewed as an expansion into the ”adjacent possible” identified by the opening network.

In order to quantify the role played by the closeness of the opening network in the learning of new openings, we considered a sample of 8831 players, forecasting the activations of new openings. We report the details about the data used in the Methods. We denote by $M_{po}^{(i)}$ the adjacency matrix obtained considering the matches played by these players in the period July 2016-September 2016 and by $M_{po}^{(f)}$ the matrix corresponding to October 2016-December 2016. In these terms, the activations *a* are those openings not played in the first period, that is $M_{pa}^{(i)}=0$. We then define the density $\rho _{pa}$ of activation *a* for player *p* as^17^

$$\begin{aligned} \rho _{pa} = \frac{\sum _o W_{ao}M_{po}^{(i)}}{\sum _o W_{ao}}, \end{aligned}$$

where $\textbf{W}$ is the adjacency matrix of the openings network previously defined. The density $\rho _{pa}$ is thus the fraction of openings connected to *a* the player *p* already used in the first time period. We define the transition probability $P(\rho )$ as the probability an activation with density $\rho$ is played in the second period considered, i.e.

$$\begin{aligned} P(\rho ) = \frac{\sum _p\sum _o\left( {1-M_{po}^{(i)}}\right) M_{po}^{(f)}\delta (\rho -\rho _{po})}{\sum _p\sum _o \left( {1-M_{po}^{(i)}}\right) \delta (\rho -\rho _{po})}. \end{aligned}$$

This quantity is plotted as function of the density in Fig. 2b. We see that the transition probability is an increasing function of the density and for large values of $\rho$ the probability for an opening to start to be played is about four times larger than at $\rho \approx 0$. This plot indicates that the density can be used to forecast the adoption of new openings. Therefore, we define the transition predictor $y^{\textrm{pred}}_{pa}$ as

3 $$\begin{aligned} {\left\{ \begin{array}{ll} y^{\textrm{pred}}_{pa} = 1 \ \text {if} \ \rho _{pa}\ge \beta \\ y^{\textrm{pred}}_{pa} = 0 \ \text {otherwise} \end{array}\right. } \end{aligned}$$

where $\beta$ is the density threshold separating openings that are predicted to be used from those that are predicted to remain unused. We tested the performance of this predictor on the activation using as ground truth the bipartite matrix of the second period $y^{\textrm{true}}_{pa}=M_{pa}^{(f)}$. Its Best F1 Score, corresponding to $\beta =0.2$, is 0.16, to be compared to Best F1 Score$=0.04$ obtained with a random predictor. We report The definition of the Best F1 Score and more details about the predictor’s performance in the Methods. This score is a good result for several reasons. First, we notice that players usually have many possible openings to start to play, but they use only a few. Second, in the similar context of the bipartite network country-product, state-of-the-art machine learning techniques reach a Best F1 Score$\approx 0.04$^14^. Moreover, here we are not interested in obtaining the best forecast possible. Instead, we want to demonstrate that our opening network contains useful information.

Concluding, we repeated the analysis described above also considering different time windows in order to understand how they affect the forecast. In particular, we obtained the following results:

- First time window: 1 month second time window: 1 month Best F1 Score: 0.13
- First time window: 6 month second time window: 3 month Best F1 Score: 0.14
- First time window: 4 month second time window: 4 month Best F1 Score: 0.15
- First time window: 2 month second time window: 2 month Best F1 Score: 0.15

In all case, we obtained performances similar to those achieved using two time windows of three months each.

**Figure 4:**
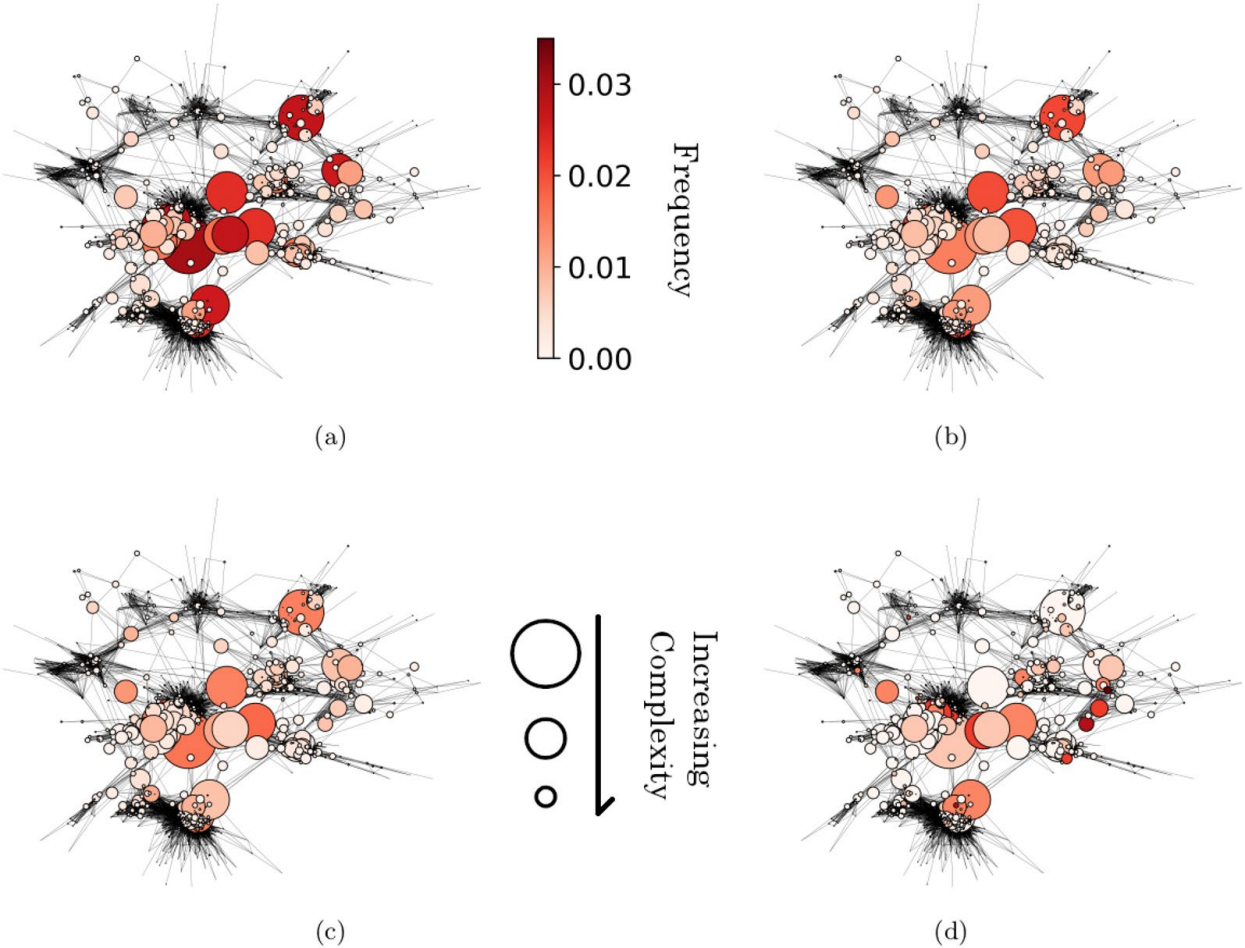
Complexity of openings. (**a**) The average opening repertoire of low-rated chess players is visualized using the opening network. We selected all players with ratings between 1500 and 1600. For each opening, we computed its frequency of use. Dark colors represent frequently used openings, while light colors represent infrequently used ones. The size of nodes is inversely proportional to the openings’ complexity, so small nodes represent hard-to-play openings, while big nodes represent easy-to-play openings. We observe that low-rated players frequently use low-complexity openings. (**b**) Same analysis for players with ratings between 1900 and 2000. (**c**) Same analysis for players with ratings between 2300 and 2400. (**d**) Same analysis for the opening repertoire of world champion Magnus Carlsen. As a player’s rating increases, low complexity openings tend to be used less frequently, while the frequency of more complex openings increases.

### The fitness of players and the complexity of openings

Not all openings are equally easy to play since some require a deep knowledge of chess theory. This is an aspect our opening network does not capture, but, as we will show, we can exploit the information contained in the player-opening bipartite network to estimate how difficult to play an opening is. First of all, we introduce the normalized matrix $\textbf{N}$ whose entries $N_{op}$ are given by the fraction of games in which player *p* used opening *o*

$$\begin{aligned} N_{op}=\frac{n_{op}}{\sum _{o}n_{op}}, \end{aligned}$$

where $n_{op}$ is the number of times player *p* chose opening *o*. This matrix defines a bipartite network of players and openings with links weighted by the frequencies of the played openings. We then exploit the Economic Fitness and Complexity (EFC) algorithm^22^ to compute the complexity of openings $Q_o$ and the fitness of player $F_p$. The former quantifies how tough to play openings are, while the latter measures the opening skills of players. The EFC algorithm is a recursive non-linear map that has been successfully applied to rank nodes of bipartite networks^22–25^ and which, in its original form, is defined by the following map

4 $$\begin{aligned} {\left\{ \begin{array}{ll} \tilde{Q}_o^{(t+1)}=\left( \sum _p N_{po}\frac{1}{F_p^{(t)}}\right) ^{-1}\\ \tilde{F}_p^{(t+1)}=\sum _o N_{po}Q_o^{(t)} \end{array}\right. } \text{ followed } \text{ by }~~~ {\left\{ \begin{array}{ll} Q_o^{(t+1)} = \frac{\tilde{Q}_o^{(t+1)}}{\langle {\tilde{Q}_o^{(t+1)}\rangle }_o}\\ F_p^{(t+1)} = \frac{\tilde{F}_p^{(t+1)}}{\langle {\tilde{F}_p^{(t+1)}\rangle }_p} \end{array}\right. } \end{aligned}$$

where by *t* we denote the iteration step. The quantities $Q_o$ and $F_p$ are given by the fixed points of such a map $Q_o=\lim _{t\rightarrow \infty }Q_o^{(t)}$ and $F_p=\lim _{t\rightarrow \infty }F_p^{(t)}$. At each time step, $Q_o^{(t+1)}$ and $F_p^{(t+1)}$ are therefore obtained by first applying the non linear map to the quantities at time *t* and then by normalizing the result (the tilde quantities) by their averages. The original map of Eq. (4) suffers of convergence issues so that we use a slightly different map, the non-homogeneous EFC (NHEFC), that delivers almost the same results but with no convergence problems^26^. We report the details of the NHEFC algorithm, its convergence and its implementation in the Methods. We add here few considerations about Eq. (4). The first expression implies that an opening has low complexity if low-fitness players play it. This is no surprise since one expects low-fitness players to use only simple-to-play openings. The second expression states that the weighted average of the complexity of openings a player uses is the player’s fitness, whereas the frequencies of the played openings determine the weights. Note that this differs from the standard EFC algorithm. In the original formulation, the fitness at the first iteration is given by the diversification $F_p^{(1)}=d_p$, while in our implementation $F_p^{(1)}=1$.

As already mentioned, openings play a significant role in chess games, so we expect players with high fitness also to have a high rating. In order to assess if this is the case, we considered a sample of 18, 253 players. We built the corresponding frequency matrix $\textbf{N}$ and we applied the NHEFC algorithm. Again, details on the data are available in the Methods. In Fig. 3a, we show the scatter plot with players’ rating against their fitness. There is a strong correlation between these two quantities, as confirmed by a Spearman correlation coefficient of 0.64. In the inset, we report the average rating as a function of the fitness (error bars defined by the standard deviation). Remarkably there are two flat regions corresponding to low-rated and high-rated players, respectively, in which an increase in the fitness does not affect the rating. This means that beginners should first learn the basic ideas of chess before focusing on opening theory. At the same time, for high-rated players, it is not very easy to improve only by focusing on openings since other aspects, such as endgames or time management, also start to be very relevant. We then repeated the Fitness analysis also considering different time controls. What we observe is that for faster games (Bullet), the correlation between the Fitness and the score of players is significantly lower (0.30), while for longer ones (Classical) the correlation is 0.55, thus not very far from the 0.64 obtained with the Blitz condition. These results confirm the common sense that Bullet games are mostly dominated by the ability to move fast, while knowledge of opening theory plays a minor role. Instead for Classical games opening theory is quite important, even if less than in Blitz games because players have sufficient time to figure out also those openings they are not very familiar with.

Going on, we studied the complexity’s meaningfulness by analyzing how players’ opening repertoire changes depending on their rating. This is done in Fig. 4a, b, c, where we considered players with rating in the ranges $1500-1600$, $1900-2000$ and $2300-2400$ and we plotted their average opening repertoire on the opening network. The sizes of nodes are inversely proportional to their complexity so that easy-to-play openings are represented as large circles, while dark colours indicate frequently used openings. We see that the opening repertoire of low-rated players is concentrated mainly on low-complexity openings, that are less frequently used by high-rated players. Analogously, Fig. 4d shows the opening repertoire of world champion Magnus Carlsen (nickname DrNykterstein): here, some of the less complex openings are completely absent, while several small nodes, corresponding to more complex openings, are dark and so frequently used. This confirms that complexity is a good indicator to quantify the difficulty of openings.

As a final test, we exploited a different classification of openings, using the *n*-plies instead of the ECO codes. A ply is defined as an “half move”, i.e. after 10 moves, 20 plies have been completed, 10 by White, 10 by Black. For a given game, we thus cut it to the first *n* half moves, so to obtain the corresponding *n*-ply. We then linked players to the *n*-plies they use as done in the case of the ECO codes. In this way we built a bipartite network of players and *n*-plies instead of the player-ECO code network we analyzed above, on top of which we can run the EFC algorithm. We show in Fig. 3b a comparison between the Fitness we obtained with such a procedure and the rating of players (the figure correspond to 6-plies). As it is possible to see, there is no correlation at all between the two measure, a situation radically different from the case where we exploited ECO codes. The same happens considering different values of *n* (we investigated up to the 8-plies) and also using only the most common *n*-plies instead of all of them. This result suggests that the plies are not suitable to efficiently disentangle between different openings, while the ECO codes, compiled by chess experts, contain much more information. The inefficiency of the *n*-plies representation of openings could be explained in terms of their distribution density. As proven in^27^, the most efficient representation of a set of items is the one showing a perfect Zipf’s law with exponent one, while, as shown in^2^, the distribution of the *n*-plies is not characterized by such an exponent. Conversely, the distribution of ECO codes shows a Zipf’s exponent close to one.

## Discussion

Chess is arguably one of the most captivating board games, and despite its ancient origins, it still enjoys a massive following around the world. One of the most intricate aspects of this game is Opening Theory, which demands years of hard work and experience to achieve mastery. Consequently, amateurs possess only a rudimentary understanding of chess openings. However, as demonstrated in this study, it is possible to extract knowledge about the Opening Theory that goes far beyond individual players’ knowledge by analyzing an entire online chess community. This approach facilitates the analysis of various aspects of chess and helps answer questions that would typically require assistance from chess experts.

As a first step, we use the player-opening bipartite network to derive the one-layer relatedness network of chess openings. The opening network quantifies the distance among chess openings, with two openings connected in this network being similar to play. Applying a community detection algorithm to the network allows us to identify ten clusters, three of which contain openings viewed from White’s perspective and correspond almost perfectly to White’s three primary options for the first move (1. e4, 1. d4, 1.c4). The remaining seven clusters correspond to Black’s most common responses to White’s first move. This structure is significant and not directly evident from the Encyclopedia of Chess Openings (ECO) classification, demonstrating that our entirely data-driven, network-based approach uncovers hidden similarities between chess openings.

We then leverage the opening network to predict which openings a player will likely start playing in the future. Our prediction relies on the assumption that openings surrounded by the player’s already used openings should be easy to learn since they are similar to what the player already knows. We introduce density, which measures the “surroundedness” of openings, to quantify the probability of an opening being played, showing that the probability of an opening being used is an increasing function of this quantity. Forecasts based on the density achieve an F1 score four times higher than those obtained by a random predictor.

Finally, we use a variation of the Economic Fitness and Complexity algorithm to develop a data-driven definition of openings’ complexity and players’ fitness. The latter quantifies players’ skill in openings and exhibits a 0.64 correlation with their rating. The former measures the level of difficulty in playing openings, and we demonstrate its relevance by showing that low-rated players tend to focus on low-complexity openings, while high-rated players also use complex openings.

The approach we used in our work considers the bipartite network of players and openings, rather than analyzing the moves contained in the openings. This represents a novel perspective on the problem, as previous works have mainly focused on the tree-like structure that arises from the presence of common moves, as first analyzed in Ref.^2^. The latter provides a somewhat complementary view of the problem: in our case, two openings are connected because many players play both of them, more often than would be expected by chance, whereas in the tree-game approach, openings are connected based on common plies, which contain the same initial sequences of moves. It would be highly interesting to compare the two methodologies and, in particular, to examine the differences and similarities of the resulting opening communities. One could also utilize the tree of moves to predict which openings a player will start to use, as it is reasonable to assume that different variations are learned by following the links of the tree corresponding to more popular openings. This approach could be combined with the one discussed in the present paper to improve the forecasting performance, since the two methodologies are likely to capture different aspects of openings learning.

We conclude by emphasizing that the analysis discussed in this paper paves the way for devising personalized recommendations for chess players. By utilizing the opening network, it is feasible to suggest to players which openings they can learn with ease. Furthermore, taking into account their fitness and the complexity of openings, such recommendations can be tailored based on players’ skill level. Additionally, the opening network combined with the complexity provides a single image that visualizes the opening repertoire of a player. This enables identifying weaknesses in opening preparation or easily comparing two players. These tools can be helpful not only to scientists interested in studying the game of chess but also to any chess player who wants to improve their opening repertoire. It is worth noting that while our work focuses on chess, the methodology we introduce is general and can be applied to any game where a bipartite network of players and openings/strategies/first moves can be defined. Examples include all chess variants (e.g., Atomic or King Of The Hill), the Japanese chess Shogi, the game of GO, or even games with *incomplete information* such as Stratego.

## Methods

### Data

We used data gathered from the online chess platform lichess.org for carrying out our analysis. These data are freely available at https://database.lichess.org/.

#### Definition of Blitz games

We only selected Blitz games since this format is the most played online. Two integer numbers define the time control of one player, e.g., “X+Y”, where X is the clock initial time in minutes and Y is the clock increment in seconds. Lichess considers a game to fall in the Blitz category if the estimated time of an average match, that is supposed to end in 40 moves per player, $T=X\times 60+40\times Y$ in seconds per player is such that $179\le T < 479$. For instance, the estimated duration of a “5+4” game is $5 \times 60 + 40 \times 4 = 460$ seconds for each player, so that “5+4” games belong to the Blitz category.

#### Players’ strength

Lichess estimates the ability of players with the *Glicko-2* rating system^28^. After each rated match, the Glicko-2 value of both players changes according to the result of the match. Generally, who win get their Glicko-2 value increased. Therefore, the higher the Glicko-2 value, the more skilled are the players. Many gaming platforms use the Glicko-2 system. With respect to the ELO system adopted by the International Chess Federation (FIDE – from the French translation –), Glicko-2 takes into account confidence intervals, i.e., an uncertainty in the assigned rating.

#### List of chess openings

We collect the list of 500 chess openings with their ECO code according to the “Encyclopaedia Of Chess Openings”^10,16^. A comprehensive list of openings can be extracted from https://en.wikipedia.org/wiki/List_of_chess_openings. We consider each opening from the point of view of both the White player and Black player, so that, in fact, the number of total openings in our analysis is 1,000.

#### Bipartite network and relatedness network

In order to build the bipartite network we used blitz games played from October 2015 to September 2016 and we applied the following filtering procedure:

- We selected only players with rating above 2000 because we expect low rated player to use some openings just by chance; considering also them would add noise to the projected opening network;
- We selected the games where the rating of the two players differs at most by 50, since if the difference between the two players is very high, then the high rated player could use a bad opening just to have more fun;
- We removed players playing less than 100 games with White and 100 games with Black so to have for each player a large statistic.

We ended up with a total of 472, 183 games involving 2513 players and 982 different openings.

#### Forecast

In order to forecast the use of openings we used two different time periods. We used blitz game data from July 2016 to September 2016 to compute the density, while the goodness of predictions have been assessed using data ranging from October 2016 to December 2016. Note that this last period does not overlap with that considered in the building of the opening network, this being an important point in order to reliably assess the goodness of the predictions. Also in this case, we retained only games with a maximum rating difference of 50 and we selected only players who did 50 matches with the White pieces and 50 games with the Black ones, so to have a large enough statistic for computing the density, ending up with a total of 88, 31 players.

#### Fitness and complexity

The fitness of players and the complexity of openings has been obtained using games played in the period October 2015 to September 2016. Differently from what done for building the bipartite network, we considered all ratings, but we made the same filtering with respect to the rating difference and the number of games played. This gives us a total of 3, 746, 135 games played between 18, 253 players who used 988 different openings. We also used the Lichess Elite Database https://database.nikonoel.fr/ to get 138 games played by Magnus Carlsen in November 2021.

### Validation of projected networks

In order to build the relatedness network of chess openings we project the bipartite matrix $M_{po}$ connecting players to openings. As explained in the main text, this can be done by using Eq. (1), that is

$$\begin{aligned} W^*_{o_1 o_2}=\sum _p^P M_{p o_1}M_{p o_2}. \end{aligned}$$

In this way, we obtain the matrix $\mathbf {W^*}$ connecting those openings appearing together in the opening repertoires of many different players. However, such a matrix is generally almost fully connected due to spurious co-occurrences. Consequently, one has to use a null model to retain only statistically significant links and filter out the spurious ones. In this work, we exploit the Bipartite Configuration Model (BiCM)^18,29,30^, based on the theory of exponential random graph; in particular we used the python bicm library https://bipartite-configuration-model.readthedocs.io/en/latest/. The BiCM is based on a canonical ensemble of random graphs defined by constraining (on average) the degree sequences of both node sets (so ubiquity and diversification). We then obtain the probability distribution of such an ensemble by maximizing Shannon entropy under this constraint. This probability distribution reads

$$\begin{aligned} P(\bar{\textbf{M}}|\{\theta _p\}, \{\mu _o\}) = \frac{{{\,\textrm{e}\,}}^{-H(\bar{\textbf{M}}|\{\theta _p\}, \{\mu _o\})}}{Z(\{\theta _p\}, \{\mu _o\})}, \end{aligned}$$

where

- $\bar{\textbf{M}}$ is the adjacency matrix of the random bipartite network
- $\{\theta _p\}$ and $\{\mu _o\}$ are the Lagrange multipliers associate respectively to the diversification of players $\{d_p\}$ and to the ubiquity of openings $\{u_o\}$
- $H(\bar{\textbf{M}}|\{\theta _p\}, \{\mu _o\})$ is the Hamiltonian, defined as
  $$\begin{aligned} H(\bar{\textbf{M}}|\{\theta _p\}, \{\mu _o\})= \sum _p \theta _p d_p(\bar{\textbf{M}}) + \sum _o \mu _o u_o(\bar{\textbf{M}}). \end{aligned}$$
- $Z(\{\theta _p\}, \{\mu _o\})$ is the partition function of the Hamiltonian
  $$\begin{aligned} Z(\{\theta _p\}, \{\mu _o\})=\sum _{\bar{\textbf{M}}}{{\,\textrm{e}\,}}^{-H(\bar{\textbf{M}}|\{\theta _p\}, \{\mu _o\})}. \end{aligned}$$

It can be shown that the probability distribution factorizes and the probability that for player *p* and opening *o* to be connected in the random network is

$$\begin{aligned} p_{po} = \frac{1}{{{\,\textrm{e}\,}}^{\theta _p+\mu _o}+1}, \end{aligned}$$

while the numerical values of the Lagrange multipliers are obtained by solving the system

$$\begin{aligned} {\left\{ \begin{array}{ll} d_p = \sum _o p_{po}\\ u_o = \sum _p p_{po}. \end{array}\right. } \end{aligned}$$

Once we have the probability of the links we can generate *N* random bipartite networks and, for each of them, compute the projected matrix $\bar{\mathbf {W^*}}$. At this point, we validate the links of $\mathbf {W^*}$, setting to one all those links which are in $99\%$ of the cases larger than the corresponding link in the random matrices. We set to zero the rest of the links. In this way, we obtain the validated matrix $\textbf{W}$ defined in Eq. (2). Here the threshold $99\%$ is arbitrary and sets the confidence level.

### Goodness of prediction test

Using the density measure we introduced above, it is possible to predict if a player will start to use a certain opening. Here we discuss about how to evaluate the goodness of these predictions. We recall that our predictions, denoted by $y^{\textrm{pred}}_{pa}$, are defined by Eq. (3)

$$\begin{aligned} {\left\{ \begin{array}{ll} y^{\textrm{pred}}_{pa} = 1 \ \text {if} \ \rho _{pa}\ge \beta \\ y^{\textrm{pred}}_{pa} = 0 \ \text {otherwise} \end{array}\right. } \end{aligned}$$

and are obtained using data in the period July 2016-September 2016, while the ground truth is obtained from data in the period October 2016-December 2016 and is defined as $y^{\textrm{true}}_{pa}=M_{pa}^{(f)}$. We recall that by *a* we denote the activations, so those openings not used by player *p* during the first period; $\beta$ is the density threshold separating the openings that we predict will be played from those that we predict will not. The most common metrics to evaluate the goodness of predictions are^31^:

- *Precision* defined as the ratio between true positives and positives (true positives plus false positives). In our case is the ratio between the number of activations we correctly predict to be played in the second period and the total number of activations we predict to be started to play. High Precision means that openings that are predicted to be played are often played in the second period;
- *Recall* given by the ratio of true positives and the sum of true positives and false negatives. A high recall implies that openings that are predicted not to be played are rarely played in the second period;
- *F1 Score* defined as the harmonic mean of Precision and Recall. The F1 score is particularly suitable when the data are unbalanced, meaning that there are many more negatives than positives (or vice versa). A high F1 Score thus implies that both the Precision and the Recall are also high.

All these indicators depend on the threshold $\beta$. We then compute the *Best F1 Score* using the threshold $\beta$ that maximizes the F1 Score, that is

$$\begin{aligned} \text {Best F1 Score}=\max _{\beta }[{\text {F1 Score}(\beta )}]. \end{aligned}$$

In the case under consideration the best threshold is $\beta =0.2$. Using this threshold we obtain

- Precision 0.10
- Recall 0.47
- Best F1 Score 0.16

### The economic fitness and complexity algorithm

The Economic Fitness and Complexity (EFC) algorithm^22,32^ is an iterative non-linear map initially designed to study the Country-Product bipartite network. It allows to compute the fitness, that is an indicator of the manufacturing capabilities of a country, and the complexity, quantifying how sophisticated and challenging it is to produce a good. This approach outperforms other techniques and allows to obtain state-of-the-art long-term GDP forecasts^12^. In our study, we apply this algorithm to the player-opening bipartite network, thus associating to each player *p* a fitness $F_p$. The higher the fitness, the more challenging openings the player plays. To each opening *p* we associate a complexity $Q_p$, that measures how difficult is that opening to play. These quantities, as mentioned above, are defined through a non-linear map given by Eqs. (4).

The iteration of Eqs. (4) leads to a fixed point which has been proved to be stable and non-dependent on initial conditions^33^. However, the EFC algorithm as defined above, in some situations has convergence issues (e.g., if the *M* matrix is not triangular shaped), so we decided to follow the Servedio et al. approach^26^ to estimate players’ fitness and the complexity of openings. Such implementation of the EFC methodology solves the convergence issues, while providing results that are highly correlated to those returned by the standard method. Instead of Eqs. (4) we use the non-homogeneous map

$$\begin{aligned} {\left\{ \begin{array}{ll} P_o^{(t+1)}=1+\sum _p \frac{N_{po}}{F_p^{(t)}}\\ F_p^{(t+1)}=\delta ^2+\sum _o \frac{N_{po}}{P_o^{(t)}} \end{array}\right. } \end{aligned}$$

where $\delta$ is a parameter that can be taken arbitrarily small. The parameter $\delta$ was introduced to take into account the basic capabilities of countries that do not export a given product. In the original method of Eq. 4, in fact, if a country do not export a good, they have zero capabilities to that regard. The same applies to products, where the term $\delta$ accounts for a minimal complexity of products. Translated to our chess world, $\delta$ models both the minimal skill that players have with respect to a given opening, and the minimal complexity of openings themselves. It also has the benefit to change the original algorithm to a non homogeneous one, healing its convergency issues. For our purposes $\delta$ can be taken small so that the algorithm converges and the final quantities are close to the original ones; here we use $\delta = 10^{-3}$. We denote by $P_o$ and $F_p$ the fixed point of the map, in these terms the complexity of openings $Q_o$ is recovered as

$$\begin{aligned} Q_o = \frac{1}{P_o-1} \end{aligned}$$

while the fitness of players are simply given by $F_p$.

## Data Availability

The datasets analysed during the current study are available in the lichess repository https://database.lichess.org/.
